# Prediction of cardiovascular risk in patients with hepatocellular carcinoma receiving anti-angiogenic drugs: lessons from sorafenib

**DOI:** 10.1007/s11739-024-03578-8

**Published:** 2024-03-29

**Authors:** Bernardo Stefanini, Francesco Tovoli, Franco Trevisani, Mariarosaria Marseglia, Giovan Giuseppe Di Costanzo, Giuseppe Cabibbo, Rodolfo Sacco, Filippo Pellizzaro, Tiziana Pressiani, Rusi Chen, Francesca Romana Ponziani, Francesco Giuseppe Foschi, Giulia Magini, Alessandro Granito, Fabio Piscaglia, Italian Liver Cancer (ITA.LI.CA) group

**Affiliations:** 1 Department of Medical and Surgical Sciences, University of Bologna, 40138 Bologna, Italy; 2 Division of Internal Medicine, Hepatobiliary and Immunoallergic Diseases, IRCCS Azienda Ospedaliero-Universitaria Di Bologna, Bologna, Italy; 3 Unit of Semeiotics, Liver and Alcohol-Related Diseases, IRCCS Azienda Ospedaliero-Universitaria Di Bologna, Bologna, Italy; 4 Liver Unit, Department of Transplantation, Cardarelli Hospital, Naples, Italy; 5 Department of Health Promotion, Mother & Child Care, Internal Medicine & Medical Specialties, PROMISE, Gastroenterology & Hepatology Unit, University of Palermo, Palermo, Italy; 6 Gastroenterology Unit, Azienda Ospedaliero-Universitaria Pisana, Pisa, Italy; 7 Gastroenterology and Digestive Endoscopy Unit, Foggia University Hospital, Foggia, Italy; 8 Department of Surgery, Oncology and Gastroenterology, Gastroenterology Unit, University of Padova, Padua, Italy; 9 Medical Oncology and Hematology Unit, Humanitas Clinical and Research Center, Rozzano (Milan), Italy; 10 Department of Specialty and Transplant Medicine, Internal Medicine and Gastroenterology, Gastroenterology, Hepatology and Transplant Unit, Fondazione Policlinico Universitario Agostino Gemelli, IRCCS, Roma- Università Cattolica del Sacro Cuore, Rome, Italy; 11 Department of Internal Medicine, Ospedale Per Gli Infermi Di Faenza, Faenza, Italy; 12 Azienda Socio Sanitaria Territoriale Papa Giovanni XXIII, Bergamo, Italy

**Keywords:** Cardiovascular events, Antiangiogenics, Sorafenib, Bevacizumab, Hepatocellular carcinoma, Stroke, Myocardial infarction, Heart failure

## Abstract

Antiangiogenics are associated with an increased risk of major adverse cardiac and cerebrovascular events (MACE). The identification of at-risk subjects is relevant in the case of hepatocellular carcinoma (HCC), for which anti-angiogenic TKIs and bevacizumab are used in first and subsequent lines of therapy, to select alternative drugs for patients with excessive risk. We verified the ability to predict MACE in sorafenib-treated patients of the 2022 European Society of Cardiology (ESC-2022) score for anti-angiogenics and the recently proposed CARDIOSOR score. A retrospective analysis was conducted of prospectively collected data of the ARPES and ITA.LI.CA databases. All patients received sorafenib for unresectable HCC from 2008 to 2018. Baseline information to calculate the ESC-2022 and CARDIOSOR scores and registration of evolutive events (including MACE) were available for all patients. The predictive ability of both scores was verified using competing risk regressions and tests for goodness of fit. This study included 843 patients (median follow-up 11.3 months). Thirty-four (4.0%) patients presented a MACE. The four-tier ESC-2022 classification showed a progressive risk increase for every class (cumulative risk 1.7%, 2.7%, 4.3%, and 15.0% in the low, medium, high, and high-risk tiers, respectively). The dichotomous CARDIOSOR scale identified a high-risk group with a fourfold increased risk of MACE (sHR 4.66, p = 0.010; cumulative risk 3.8% and 16.4%). ESC-2022 showed a better goodness of fit compared to the CARDIOSOR score [C-index 0.671 (0.583–0.758) vs 0.562 (0.501–0.634), *p* = 0.021], but this gap was eliminated using the linear version of CARDIOSOR. Both the ESC-2022 and CARDIOSOR scores discriminated patients at increased risk for MACE. The use of these scores in clinical practice should be encouraged, since therapeutic measures can mitigate the cardiovascular risk.

**Supplementary Information:**

The online version contains supplementary material available at 10.1007/s11739-024-03578-8.

## Introduction

Hepatocellular carcinoma (HCC) is a leading global cause of death [1]. Its prevalence is rising due to the aging population and a metabolic dysfunction-associated steatotic liver disease (MASLD) epidemic [2, 3]. Over 50% of HCC patients undergo systemic treatment as part of its natural history [4].

Tumor angiogenesis inhibition is a cornerstone of pharmacological treatment for HCC, aiming for tumor vascular normalization and a resolution of hypoxia in tumor microenvironment [5, 6].

Inhibition of the angiogenesis can be achieved through monoclonal antibodies against circulating vascular endothelial growth factor (VEGF) or through tyrosine kinase inhibitors (TKI) targeting the VEGF receptor (VEGFR).

Sorafenib was the first TKI showing survival benefits in HCC patients [7]. In 2020, the combination of the immune checkpoint inhibitor (ICI) atezolizumab and the anti-VEGF antibody bevacizumab demonstrated superiority to sorafenib [8], becoming the new standard of care. Other TKIs, such as lenvatinib [9], regorafenib [10] and cabozantinib [11], are used in subsequent therapeutic lines, with ongoing exploration of novel combination strategies [12].

Unfortunately, anti-angiogenic therapies are also linked to an array of cardiovascular adverse events, mainly due to the role of the VEGF pathway in physiological pro-angiogenic processes and metabolic homeostasis [13]. Major adverse cardiovascular events (MACE) have been reported, often leading to permanent drug discontinuation and impaired quality of life [14]. These events include hearth failure, acute coronary syndrome, cerebrovascular accidents, and peripheral limb ischemia [14].

The European Society of Cardiology (ESC) recently ###updated its clinical guideline on cardio-oncology [13]. A dedicated tool, different from the conventional cardiovascular risk calculators was developed. In fact, both cancer itself and anticancer therapies can increase the likelihood of cardiovascular diseases [15, 16]. These guidelines consider anti-VEGF antibodies and TKIs as a single category, with dedicated recommendations for a general approach, cardiovascular risk evaluation, prevention, and monitoring [13]. To the best of our knowledge, the performance of the ESC cardio-oncology risk stratification has never been explored in HCC patients on anti-angiogenic therapy.

Different from other malignancies, HCC often arises in the setting of liver cirrhosis, a condition associated with reduced cardiac performance [17] and hypercoagulability [18].

Consequently, the CARDIOSOR score has been proposed as an alternative possible tool to evaluate the cardiovascular risk in HCC patients candidates for sorafenib [19]. However, its performance was not compared with the ESC score nor supported by an external validation.

This study compared the prognostic accuracy of ESC and CARDIOSOR scores in a large cohort of HCC sorafenib-treated HCC patients. We selected sorafenib as it currently offers the advantage of recruiting the largest number of treated patients and the longest follow-up.

## Methods

### Study population

This study was performed using the medical records from two databases: the Archives of Patients with hEpatocellular carcinoma treated with Sorafenib (ARPES), and the Italian Liver Cancer registry (ITA.LI.CA). Both databases include patients consecutively diagnosed with HCC and treated in different hospitals throughout Italy. Participating centres collected data prospectively and updated them every 3–6 months for the ARPES and every two years for the ITA.LI.CA registry.

For this study, we evaluated all patients in the ARPES database and patients in the ITA.LI.CA registry who received sorafenib between January 2010 and December 2018. The starting date coincides with the creation of the ARPES database. The closing date was chosen to allow an adequate follow-up for all patients. The last follow-up examinations were performed in September 2022.

### Baseline evaluation

The following data were available for all patients at the start of sorafenib: liver function (Child–Pugh score), tumor staging according to BCLC classification, alfa-fetoprotein (AFP) level, Eastern Cooperative Oncology Group – Performance Status (ECOG-PS), medical history including cardiovascular risk factors (i.e. dyslipidaemia, hypertension, smoking status) and previous cardiovascular diseases.

### European Society of Cardiology score -2022 version

**Fig. 1:**
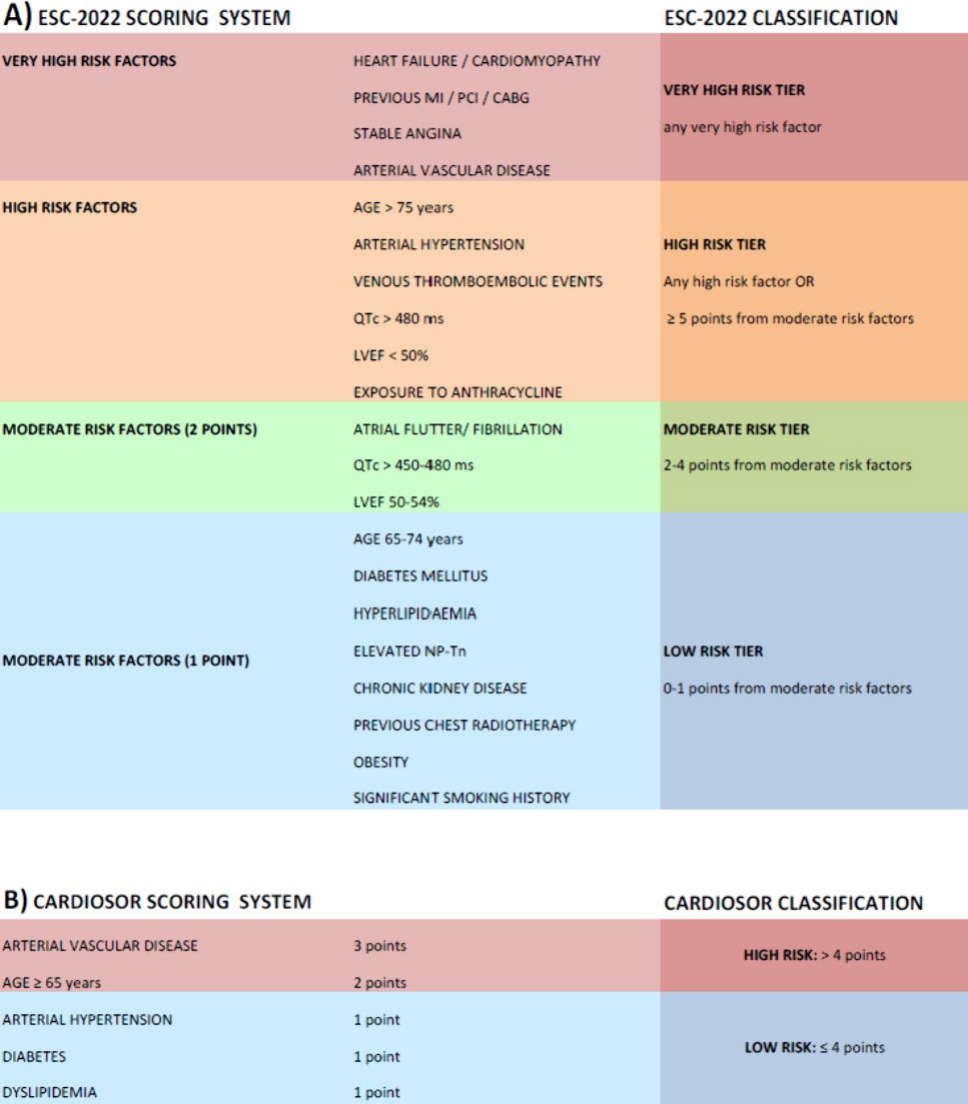
Overview of the European Society Cardio-Oncology group 2022 (ESC-2022) and CARDIOSOR scores. *MI* myocardial infarction, *PCI* percutaneous coronary intervention, *CABG* coronary artery bypass graft, *LVEF* left ventricular ejection fraction, *BNP* brain natriuretic peptide, *Tn* troponin

This score was calculated following the ESC 2022 guidelines from the Cardio-Oncology Group [13] (Fig. 1). Amongst the many elements composing this score, the following items had been systematically considered for every patient before starting sorafenib as part of the standard clinical practice and following the ESC recommendations: age, previous history of cardiovascular disease (including but not limited to heart failure, history of angina, history of deep vein thrombosis or pulmonary embolism), generic cardiovascular risk factors (including hypertension, chronic kidney disease, diabetes, hyperlipidemia), previous or current cancer treatment and lifestyle risk factors.

Additionally, cardiac evaluation by a cardiologist and pertinent tests (including cardiac markers and imaging, when appropriate) had been performed in patients with a history of cardiovascular events or multiple cardiovascular risk factors, as part of the usual clinical practice and following the ESC recommendations [13].

The ESC algorithm provided the final class of the patient. Each item of this score was assigned a degree of severity, as follows: moderate 1 (M1) equal to 1 point, moderate 2 (M2) equal to 2 points, high (H) and very high (VH). Patients with at least one VH risk factor were directly assigned to very high-risk tier; the high-risk group included either patients with at least one H factor or a sum of moderate-risk factors accounting for a ≥ 5 points; the moderate-risk tier included patients with M1-M2 risk factors and a score between 2 and 4 points. Finally, the low-risk class included only patients without VH/H factors and a score < 2.

### CARDIOSOR score

Calculating the CARDIOSOR score required the following information: age, history of hypertension, diabetes, dyslipidemia, and peripheral vascular disease. All of these data had been investigated as part of the standard clinical practice in all patients before starting sorafenib.

As proposed by Carballo-Folgoso et al. [19], patients were assigned 1 point each for hypertension, diabetes, and dyslipidemia; 2 points for age ≥ 65 years; 3 points for history of stroke, ischemic cardiac disease, peripheral vascular disease. A score of ≤ 4 points identified low-risk patients, while scores > 4 indicated a high-risk condition (Fig. 1).

### Follow-up

At the time of the first prescription of sorafenib, all patients were provided with a diary and instructed about the possible sorafenib-related AEs to allow early recognition and treatment. Amongst other measures, patients were advised to measure blood pressure daily and contact their respective centers in case of new symptoms or events. Cardiovascular and other major adverse events arising after the treatment start were thoroughly recorded on the occasion of each follow-up examination (scheduled about every 2–3 months in parallel to the tumor re-staging). Moreover, patients were contacted by phone at least every 4 weeks, as the Italian Drugs Agency (AIFA) mandates a re-evaluation of possible contra-indications to continue high-cost drugs monthly.

### Major cardiovascular adverse events

Several definitions of MACE are available in literature [13]. We adopted the same definition used in previous studies of systemic treatments for HCC [19]. In detail, MACE was defined as the occurrence of heart failure (HF) or acute coronary syndrome (ACS) according to the current European Cardiovascular Society definition, cerebrovascular accidents (CVA), or peripheral ischemia.

### Statistical analyses

Continuous variables data were described using median and interquartile range (IQR). Categorical variables are expressed as absolute and relative frequencies.

Overall survival (OS) was defined as the time from the starting dose of sorafenib until the date of death, the last available visit, or the end of the follow-up period (whichever occurred first).

Time to MACE (MACE-t) was defined as the time from the start of sorafenib until the development of MACE. Since death and development of MACE are to be considered as competing outcomes, we used a competing risk regression to verify the ability of our scores to predict the occurrence of MACE. Any cause of death apart from cardiovascular death was considered a competing event. Subdistribution hazard ratios (sHR) and their 95% confidence interval (CI) were reported for every cardiovascular risk factor and for ESC and CARDIOSOR scores. *P* values < 0.05 were considered statistically significant.

Akaike’s information criteria (AIC) [20] and Bayesian information criteria (BIC) [21] were calculated to verify the predictive ability of the scores, as per Kuk and Varadhan instructions for competing-risk regressions [22]. BIC is related to AIC, but penalizes more additional parameters than AIC, resulting in BIC favoring more parsimonious models than AIC. Lower AIC and BIC scores indicate better goodness of fit. Finally, we used the Wolbers et al. [23] adaptation of Harrell’s C-statistic to the competing risk setting, where death status is switched to censored and the time to event is equal to the longest possible time to event that any enrolled patient was followed up. Since the Wolbers’s method does not provide a comparison between different C-statistic values, the Harrell’s C-statistic was also provided and calculated assuming MACE as failure event, and by censoring deaths for any non-cardiovascular events (for instance, HCC-related deaths).

Considering the established role of viral hepatitis as a modifier of cardiovascular risk [24, 25], a sensitivity analysis was designed to include only patients with viral liver disease.

All statistical analyses were performed with STATA/SE 14.1 (StataCorp), using the “stcrreg”, “stcompet”, and “somerd” syntax.

### Ethics

The present study was conducted following the ethical guidelines of the Declaration of Helsinki. The data collection into the ARPES and ITA.LI.CA registries was approved by the Independent Ethic Committee of the IRCCS Azienda Ospedaliero-Universitaria of Bologna (protocols n. 098/2014/OSS and 99/2012/O/Oss, respectively) that operated as coordinating centers of both networks. In all the remaining centers, data inclusion into the registries was approved by the local ethics committees. All patients provided a written informed consent allowing their data to be conserved with an anonymized identification number. Both the ITA.LI.CA and the ARPES database conform to the current European Union Regulations on privacy.

## Results

### Study population

**Fig. 2:**
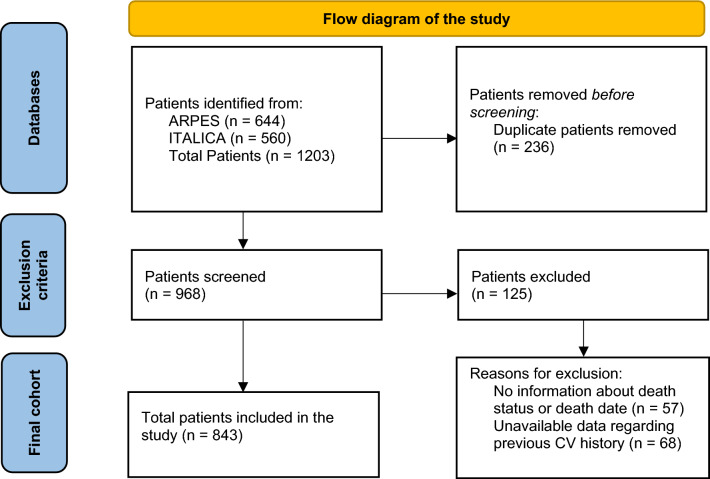
Flow diagram of the study

This study included 843 patients (Fig. 2). Most of our patients were cirrhotic with a predominance of viral etiology and male sex (Table 1). The median overall survival was 10.2 months (CI 95% 9.3–11.1 months) and the median follow-up was 11.3 months (CI 95% 10.1–12.3 months).

**Table 1:** Baseline characteristics of the patients in the whole study cohort and across the different risk classes of the European Society of Cardiology score

Variables	Whole cohort (*n* = 842)	Low-risk ESC (*n* = 265)	Moderate-risk ESC (*n* = 82)	High-risk ESC (*n* = 400)	Very-high-risk ESC (*n* = 95)
Age (years)	68 (60–74)	60 (53–67)	69 (66–71)	72 (65–78)	73 (68–78)
Sex (male)	701 (83.3)	228 (86.0)	66 (80.5)	322 (80.6)	86 (90.5)
Viral etiology	597 (70.9)	568 (72.4)	49 (59.7)	275 (68.8)	54 (56.8)
HCV	441 (52.4)	141 (53.2)	43 (52.4)	214 (53.5)	43 (45.3)
HBV	178 (21.1)	90 (34.0)	6 (7.3)	70 (17.5)	12 (12.6)
HDV + HBV	16 (1.9)	9 (3.4)	0 (0.0)	6 (1.5)	1 (1.1)
Non-viral HCC	245 (29.1)	45 (17.0)	33 (40.3)	125 (31.3)	41 (43.2)
Cirrhosis (present)	785 (93.2)	244 (92.1)	76 (92.7)	380 (95.0)	84 (88.4)
*BCLC*					
A Early	22 (2.6)	7 (2.6)	4 (4.9)	8 (2.0)	3 (3.2)
B Intermediate	119 (14.1)	29 (10.9)	16 (19.5)	58 (14.5)	16 (16.8)
C Advanced	690 (82.0)	223 (84.2)	59 (72.0)	333 (83.0)	76 (80.0)
D Terminal	11 (1.3)	6 (2.3)	3 (3.7)	2 (0.5)	0 (0.0)
*Child–Pugh class*					
A	668 (81.9)	216 (81.5)	60 (73.2)	340 (85.0)	73 (76.9)
B	141 (17.3)	44 (16.6)	21 (25.6)	58 (14.5)	22 (23.2)
C	6 (0.7)	3 (1.1)	1 (1.2)	2 (0.5)	0 (0.0)
Neoplastic PVT	391 (46.4)	138 (52.1)	40 (48.8)	178 (44.6)	36 (37.9)
MELD	9 (8–11)	9 (8–11)	9 (8–12)	9 (7–11)	9 (8–11)
Hypertension	382 (45.4)	0 (0.0)	0 (0.0)	322 (80.5)	60 (63.2)
Diabetes	255 (30.3)	21 (7.9)	53 (64.6)	139 (34.8)	42 (44.2)
Dyslipidemia	59 (7.0)	2 (0.8)	10 (12.2)	28 (7.0)	19 (20.0)
CIC	81 (9.6)	0 (0.0)	0 (0.0)	0 (0,0)	81 (85.3)
Chronic kidney disease	32 (3.8%)	1 (0.4)	5 (6.1)	17 (4,3)	9 (9.5)
Obesity (BMI ≥ 30)	72 (8.6)	8 (3.0)	16 (19.5)	36 (9.0)	12 (12.6)
Smoker	135 (16.0)	19 (7.2)	35 (42.7)	68 (17.0)	11 (11.6)
Peripheral arterial occlusive disease	19 (2.2)	0 (0.0)	0 (0.0)	0 (0.0)	19 (20.0)

At the start of sorafenib, hypertension was the most frequent cardiovascular risk factor (44.4%), followed by diabetes (30.1%) and smoking habit (15.2%); other well-known cardiovascular risk factors, such as ischemic heart disease, chronic kidney damage, obesity, and peripheral arterial occlusive disease were less prevalent.

### Distribution of the population according to the CV risk scores

About half of the patients (*n* = 401, 47.6%) were classified as high risk according to the ESC classification. A lower proportion was included in the low-risk (*n* = 264, 31.3%), very-high (*n* = 95, 11.3%), and moderate classes (*n* = 83, 9.8%). According to the CARDIOSOR score, 805 (95.5%) and 38 (4.5%) patients were categorized as low risk and high risk, respectively (Supplementary Figure S1).

### Treatment-emergent MACE

During the follow-up, 34 (4.0%) patients suffered a MACE. The median time to MACE was 111 days (range 5–1123). The most frequent events were CVA (*n* = 15), ACS (*n* = 12), and HF (*n* = 7).

These events were fatal in 19 patients and required hospitalization in the remaining patients. Among patients who survived MACE, sorafenib was permanently discontinued (*n* = 8), temporarily discontinued, and restarted at the same (*n* = 3) or reduced dose (*n* = 4). Among patients who survived the event, the median post-MACE survival was 5.9 months (95% CI 3.8–7.9 months).

Analyzing the role of each single risk factor, only ischemic heart disease (sHR 5.275, 95% CI 2.552–10.903, *p* < 0.001) and peripheral arterial occlusive disease (sHR 6.181, 95% CI 2.229–17.136, *p* < 0.001) were associated with an increased risk of developing MACE. Other well-known risk factors, such as diabetes, obesity, dyslipidemia and older age, showed a trend that did not reach the pre-defined statistical threshold.

Other risk factors, such as the viral etiology of cirrhosis, were not associated with increased risk of MACE (Supplementary Table S1).

### Predictive abilities of the scores

**Fig. 3:**
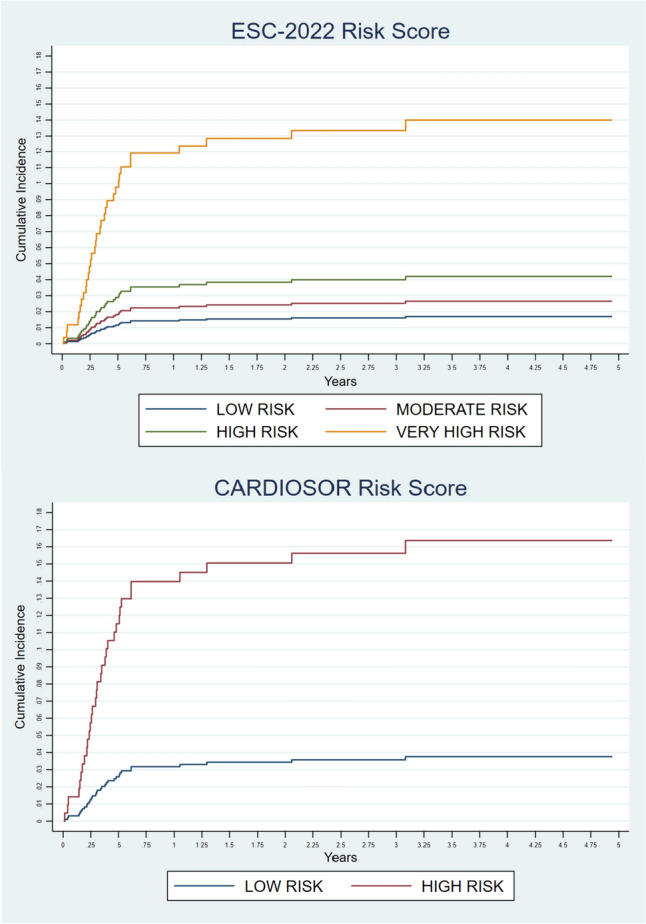
Risk of major adverse cardiovascular events according to the European Society for Cardiology 2022 (ESC-2022 —Panel A) and CARDIOSOR score (Panel B)

Both the ESC risk score and CARDIOSOR scale effectively discriminated between patients at high risk and low risk of developing MACE (Fig. 3).

According to ESC-2022 score, the cumulative rate of MACE increased from 1.7% in the low-risk group to 15% in the very high-risk group (Table 2), with an eightfold increase of the cumulative risk from the lowest to the highest risk class (sHR 8.836, 95% CI 2.840–27.482, *p* < 0.001). Dichotomizing the patients into two groups (very high risk vs other classes, the sHR lowered to 5.336 (95% CI 2.662–10.69, *p* < 0.001).

**Table 2:** Cumulative risk of major cardiovascular adverse events according to the competing risk analysis

RISK SCORE	PATIENTS (%)	EVENTS (%)	1-YEAR RISK (%)	CUMULATIVE RISK (%)	SHR	95% CI	*P*
*ESC 2022*							
LOW	259 (31.8)	4 (11.8)	1.4	1.7	REFERENCE		
MODERATE	78 (9.6)	3 (8.8)	2.3	2.7	1.575	0.287–8.648	0.601
HIGH	389 (47.7)	15 (44.1)	3.6	4.3	2.513	0.852–7.617	0.072
VERY HIGH	89 (10.9)	12 (35.3)	11.9	15.0	8.836	2.840–27.482	< 0.001
*CARDIOSOR*							
≤ 4 POINTS (LOW)	784 (96.2)	28 (82.4)	3.2	3.8	REFERENCE		
> 4 POINTS (HIGH)	31 (3.8)	6 (17.6)	14.0	16.4	4.666	1.969–11-059	< 0.001
*ESC 2022*							
VERY HIGH	89 (10.9)	12 (35.3)	12.8	15.0	5.336	2.662–10.696	< 0.001
OTHER	726 (89.1)	22 (64.7)	2.5	3.0	REFERENCE		
**CARDIOSOR (LINEAR)**	843(100)	34 (100)	NA	NA	1.431	1.171–1.751	< 0.001

*sHR* subdistribution hazard ratio, *CI* confidence interval, *ESC* European Society of Cardiology

According to the CARDIOSOR scale, low-risk patients had a cumulative rate of MACE of 3.8%, while in the high-risk group the percentage increased to 16.4%, accounting for a fourfold increase in the risk of developing MACE (sHR 4.666, 95% CI 1.969–11.059, *p* < 0.001). When the CARDIOSOR scale was explored as a linear predictor of the risk of MACE, the risk grew as the points accumulated, with a linear sHR = 1.431 per point (95% CI 1.171–1.751, *p* < 0.001).

The two scores had very similar goodness of fit as defined by the Bayesian and Akaike information criteria (ESC-2022: BIC = 366, AIC = 361, CARDIOSOR: BIC = 371, AIC = 366). Instead, the C-index analyses showed that the ESC-2022 score outperformed the CARDIOSOR score [Wolbers C-index 0.671 (0.583–0.758) vs 0.562 (0.501–0.634), *p* = 0.021]. However, when the CARDIOSOR score was examined as a linear predictor, its performance was not significantly different from that of the ESC-2022 score (Table 3).

**Table 3:** Accuracy of the European Society of Cardiology (ESC-2022) score compared to the CARDIOSOR score

Prognostic score	Harrell’s c-index (95%CI)	*P* (Harrell)	Wolbers c-index (95%CI)
*ESC-2022*			
- 4 classes	0.654 (0.560–0.747)	–	0.671 (0.583–0.758)
- 2 classes	0.623 (0.541–0.706)	0.353	0.634 (0.554–0.715)
*CARDIOSOR*			
- 2 classes	0.551 (0.494–0.609)	0.021	0.562 (0.501–0.634)
- Linear predictor	0.630 (0.533–0.726)	0.502	0.645 (0.553–0.740)

NOTE. Accuracy is calculated according to MACE-free specific survival (Harrell) and competing-risk survival (Wolbers). *P* values refer to comparison of Harrell’s c-statistics between the ESC-2022 four-class model and the other risk scores

### Sensitivity analysis

The sensitivity analysis included 597 patients with viral etiology, 22 of whom experienced a MACE. In this analysis, the ESC-2022 very-high risk group maintained its predictive abilities (sHR 7.346, 95% CI 2.176–24.799, *p* < 0.001) versus the low-risk group. The CARDIOSOR linear predictor also preserved its prognostic abilities (sHR 1.542, 95% CI 1.092–2.178, *p* = 0.014). Conversely, the dichotomized CARDIOSOR score maintained a median sHR value similar to that of the whole study population, but with wider confidence intervals due to the loss of some events, and a consequent loss of full statistical significance (sHR 4.061, 95% CI 0.670–24.734, *p* = 0.128). Full results are displayed in Supplementary Table S2.

## Discussion

Anti-angiogenic drugs are an important part of the therapeutic armamentarium against HCC. Since severe cardiovascular toxicities can jeopardize the survival benefit offered by these drugs, identifying patients at high risk for these events is crucial to optimize the risk–benefit ratio and to tailor treatment regimens. This point will become more prominent in the future, considering the expected rise of HCC related to MASLD, a condition intrinsically at high risk of cardiovascular events [26].

Our study confirmed that the occurrence of MACE was relatively common among patients treated with sorafenib, but also showed that we dispose of practical tools to predict the risk of occurrence of these events.

In our cohort, the cumulative incidence of MACE was 4%. This figure is slightly higher compared to those found in previous studies [27, 28], but lower than the staggering value (11%) reported by Carballo-Folgoso et al. [19]. This difference can be explained by relevant dissimilarities in the baseline characteristics of the enrolled populations, especially regarding cardiovascular risk factors, including dyslipidemia (25.0% vs 6.6%), obesity (27.7% vs 8.1%), and peripheral arterial disease (7% vs 2.2%). Therefore, it appears evident that patient selection can heavily impact on the expected number of MACE during the follow-up.

Testing the performance of risk scores was the novel and most relevant part of our study. The ESC score has been proposed for cardio-oncology and has never been tested in HCC patients. Conversely, the CARDIOSOR score was explicitly developed for HCC patients receiving sorafenib, but it was not validated in an independent cohort. We found that both scores effectively identified a subgroup of patients at increased risk of MACE. Our results were largely confirmed when analyzing only patients with viral etiology.

Goodness-of-fit tests favored ESC-2022 over CARDIOR scores. This difference was expected, as the two scores have some peculiarities worth to be discussed. The ESC-2022 has an intrinsic "ceiling effect”, as all patients with a single high or very high risk factors are classified in the same category, regardless of the presence of additional risk factors. The dichotomized CARDIOSOR suffers from a similar problem. However, CARDIOSOR as linear predictor had a good performance, probably as it considered the sum of all potential risk factors. This finding suggests that CARDIOSOR should be categorized into more than two classes to improve its prognostic accuracy.

Some limitations of our study need to be discussed. First, as a real-life study, some cardiovascular information (for instance, cardiac biomarkers and ultrasound imaging to estimate left ventricular ejection fraction) were investigated only when clinically indicated (i.e., according to the ESC Cardio-Oncology recommendations). Patients without cardiovascular risk factors and asymptomatic cardiac dysfunction are probably rare. However, we cannot rule out some exceptional misclassification which could have been averted only by a dedicated prospective study in which all required parameters are checked for every patient. Second, we did not systematically registertreatment-emergent minor cardiovascular events, such as atrial fibrillation or QT interval prolongation. While treatment-emergent events are not accounted for in the examined score, the overall cardiovascular risk of patients under antineoplastic treatment is a complex and dynamic interplay. As such, the interest in these minor events cannot be discarded entirely.

The possible extension of our results to other TKIs (lenvatinib, regorafenib, cabozantinib) or anti-VEGF antibodies (bevacizumab, ramucirumab) deserves discussion. The ESC Cardio-Oncology guidelines consider all the anti-angiogenic treatments similar in terms of risk of MACE. A recent meta-analysis including 20,027 patients from 45 randomized controlled trials treated with nine FDA-approved VEGFR-TKIs (axitinib, cabozantinib, lenvatinib, nintedanib, pazopanib, regorafenib, sorafenib, sunitinib, vandetanib) [14] supports this opinion. The authors found significant differences in the prevalence of treatment-emergent minor cardiovascular toxicities (for instance, hypertension), but none in severe toxicities [14]. On these premises, it seems possible to extend our results to other TKIs. Still, we believe that specific data should be collected for anti-VEGF antibodies before extending the conclusions of our study to these drugs. Nevertheless, our results remain useful as TKIs still have a non-negligible role in the treatment of HCC, either as part of therapeutic combinations and sequences [11, 29–31] or for patients with contraindications to immunotherapy (including transplant recipients or patients with severe autoimmune diseases) [32].

High-risk patients should undergo a cardiological evaluation and adopt available strategies to reduce the probability of developing a MACE. Notably, many drugs commonly used in primary and secondary cardiovascular prevention have shown potentially positive effects in the setting of liver cirrhosis and cancer, including aspirin [33, 34], beta-adrenergic blocking agents [35], angiotensin receptor blockers [36, 37], and statins [38]. Alternatively, patients in the highest risk tiers should be considered to receive pure immunotherapy-based regimens such as the STRIDE regimen (a single dose of tremelimumab, followed by chronic administration of durvalumab) [39] or monotherapies (nivolumab, pembrolizumab, durvalumab) according to the local regulatory agencies’ approvals.

Even the dichotomization of the population with either score in a large low-risk and a small high-risk subgroups can be useful. In fact, consideration of refrain from starting treatment or using antiangiogenics-free regimens could be reserved to this limited group.

In conclusion, we found that both the ESC-2022 and CARDIOSOR scores, the latter in its linear variant, could accurately stratify the cardiovascular risk of sorafenib-treated patients. Therefore, the use of these scores should be encouraged in clinical practice. Once identified, patients with a clinically relevant cardiovascular risk should be managed either through concomitant treatment optimization or their allocation to therapeutic regimens without anti-angiogenic molecules.

### Supplementary Information

Below is the link to the electronic supplementary material.

Supplementary file1 (DOCX 51 KB)

Supplementary file2 (DOCX 22 KB)

## Data Availability

The data presented in this study are available on request from the corresponding author.
