# Supplementary material for: Prediction of cardiovascular risk in patients with hepatocellular carcinoma receiving anti-angiogenic drugs: lessons from sorafenib
Source: Intern Emerg Med. 2024 Mar 29;19(4):1151–60. doi: 10.1007/s11739-024-03578-8 (PMC11186950; doi:10.1007/s11739-024-03578-8)

**SUPPLEMENTARY FIGURE**

Figure S1. Categorisation of the study population according to the European Society for Cardiology 2022 (ESC-2022 – Panel A) and CARDIOSOR score (Panel B)


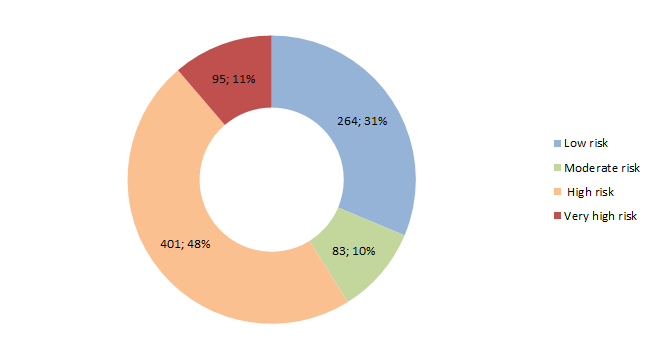


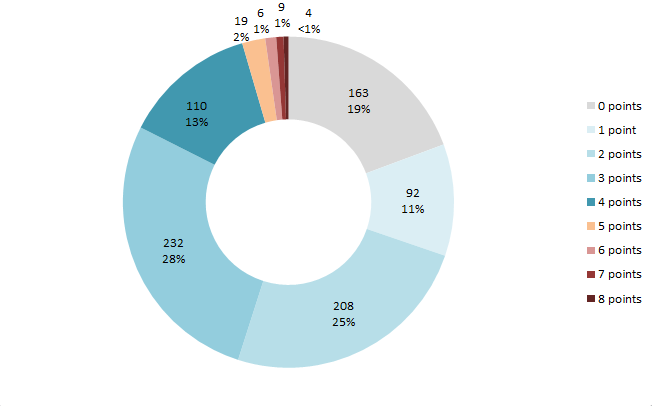

Supplement: Supplementary file 1 — Supplementary file1 (DOCX 51 KB) [file 11739_2024_3578_MOESM1_ESM.docx]
