# Supplementary material for: Prediction of cardiovascular risk in patients with hepatocellular carcinoma receiving anti-angiogenic drugs: lessons from sorafenib
Source: Intern Emerg Med. 2024 Mar 29;19(4):1151–60. doi: 10.1007/s11739-024-03578-8 (PMC11186950; doi:10.1007/s11739-024-03578-8)
Supplement: Supplementary file 2 — Supplementary file2 (DOCX 22 KB) [file 11739_2024_3578_MOESM2_ESM.docx]

**SUPPLEMENTARY TABLES**

**Table S1.** Univariable competing risk regression of developing MACE in the whole study population (n=815)

| **Variable** | ***sHR*** | ***95% CI*** | ***P*** |
| --- | --- | --- | --- |
| Age |  |  |  |
| - <65 years | Reference |  |  |
| - 65-74 years | 1.664 | 0.704-3.930 | *0.246* |
| - ≥ 75 years | 1.836 | 0.724-4.658 | *0.201* |
| Sex (male) | 1.859 | 0.563-1.635 | 0.310 |
| Hypertension | 1.509 | 0.744-3.062 | 0.254 |
| Obesity (BMI≥30) | 1.954 | 0.770-4.962 | 0.159 |
| Chronic kidney injury | 2.408 | 0.756-7.662 | 0.137 |
| Ischemic hearth disease | 5.275 | 2.552-10.903 | ***<0.001*** |
| Dyslipidemia | 1.826 | 0.650-5.124 | *0.253* |
| Diabetes | 1.622 | 0.802-3.278 | *0.179* |
| Significant smoking history | 0.926 | 0.372-2.486 | 0.936 |
| Viral etiology | 0.713 | 0.359-1.450 | 0.351 |
| HCV | 0.935 | 0.472-1.851 | 0.935 |
| Peripheral arterial occlusive disease | 6.181 | 2.229-17.136 | ***<0.001*** |

|  |  |  |  |
| --- | --- | --- | --- |

| Risk score | PATIENTS 597 (%) | Events 22 (%) | 1-year RISK (%) | cumulative risk (%) | sHR | 95% CI | p |
| --- | --- | --- | --- | --- | --- | --- | --- |
| ESC 2022 |  |  |  |  |  |  |  |
| - low | 220 (36.9) | 3 (13.6) | 1.4 | 2.2 | REFERENCE |  |  |
| - moderate | 48 (8.0) | 2 (9.1) | 2.3 | 2.3 | 1.098 | 0.287-8.648 | 0.933 |
| - high | 275 (46.1) | 9 (40.9) | 3.3 | 4.5 | 2.043 | 0.852-7.617 | 0.228 |
| - very high | 54 (9.0) | 8 (36.4) | 12.2 | 14.4 | 7.346 | 2.176-24.799 | **<0.001** |
|  |  |  |  |  |  |  |  |
| CARDIOSOR |  |  |  |  |  |  |  |
| - ≤ 4 POINTS (low) | 582 (97.4) | 18 (81.8) | 0.8 | 1.0 | REFERENCE |  |  |
| - > 4 POINTS (HIGH) | 15 (2.6) | 4 (18.2) | 3.3 | 4.8 | 4.061 | 0.670-24.734 | 0.128 |
|  |  |  |  |  |  |  |  |
| CARDIOSOR linear | 597 (100) | 22 (100) | NA | NA | 1.542 | 1.092-2.178 | **0,014** |

**Table S2.** Cumulative risk of major cardiovascular adverse events considering only patients with an underlying viral disease (n=597) according to the competing risk analysis.

sHR: subdistribution hazard ratio, CI: confidence interval; ESC: European Society of Cardiology
